# Hybrid Deep Learning Architecture with Adaptive Feature Fusion for Multi-Stage Alzheimer’s Disease Classification

**DOI:** 10.3390/brainsci15060612

**Published:** 2025-06-06

**Authors:** Ahmad Muhammad, Qi Jin, Osman Elwasila, Yonis Gulzar

**Affiliations:** 1 School of Information and Communication Engineering, University of Electronic Science and Technology of China, Chengdu 611731, China; ahmadjameel7171@gmail.com (A.M.); jqi@uestc.edu.cn (Q.J.); 2 Department of Management Information Systems, College of Business Administration, King Faisal University, Al-Ahsa 31982, Saudi Arabia

**Keywords:** Alzheimer’s disease, deep learning, convolutional neural networks, vision transformers, adaptive feature fusion, MRI classification, neuroimaging

## Abstract

Background/Objectives: Alzheimer’s disease (AD), a progressive neurodegenerative disorder, demands precise early diagnosis to enable timely interventions. Traditional convolutional neural networks (CNNs) and deep learning models often fail to effectively integrate localized brain changes with global connectivity patterns, limiting their efficacy in Alzheimer’s disease (AD) classification. Methods: This research proposes a novel deep learning framework for multi-stage Alzheimer’s disease (AD) classification using T1-weighted MRI scans. The adaptive feature fusion layer, a pivotal advancement, facilitates the dynamic integration of features extracted from a ResNet50-based CNN and a vision transformer (ViT). Unlike static fusion methods, our adaptive feature fusion layer employs an attention mechanism to dynamically integrate ResNet50’s localized structural features and vision transformer (ViT) global connectivity patterns, significantly enhancing stage-specific Alzheimer’s disease classification accuracy. Results: Evaluated on the Alzheimer’s 5-Class (AD5C) dataset comprising 2380 MRI scans, the framework achieves an accuracy of 99.42% (precision: 99.55%; recall: 99.46%; F1-score: 99.50%), surpassing the prior benchmark of 98.24% by 1.18%. Ablation studies underscore the essential role of adaptive feature fusion in minimizing misclassifications, while external validation on a four-class dataset confirms robust generalizability. Conclusions: This framework enables precise early Alzheimer’s disease (AD) diagnosis by integrating multi-scale neuroimaging features, empowering clinicians to optimize patient care through timely and targeted interventions.

## 1. Introduction

Alzheimer’s disease (AD), a progressive neurodegenerative disorder, profoundly impacts cognitive functions, memory, and behavior, imposing a substantial burden on global healthcare systems [1,2]. Its complex pathology manifests through localized structural alterations, such as hippocampal atrophy and cortical thinning, coupled with disruptions in long-range neural connectivity, which collectively drive cognitive decline [3]. Early and accurate diagnosis is critical for initiating timely interventions to mitigate disease progression; however, conventional neuroimaging techniques often fail to capture the subtle, multifaceted pathological signatures of Alzheimer’s disease (AD) [4]. T1-weighted magnetic resonance imaging (MRI) remains a cornerstone for non-invasive AD diagnosis, revealing structural abnormalities critical for staging [4]. Yet, manual interpretation of MRI scans is inherently subjective and prone to overlooking nuanced changes, necessitating advanced computational approaches to enhance diagnostic precision [5].

Deep learning has revolutionized Alzheimer’s disease (AD) diagnostics by extracting high-dimensional feature embeddings from MRI data, enabling the identification of disease-specific patterns with unprecedented accuracy [5]. Despite these advancements, many models are limited by their focus on either localized features, such as textural anomalies or regional atrophy [6], or global brain alterations, like ventricular enlargement [7], without effectively integrating these complementary perspectives [8]. This fragmented approach hampers generalization across diverse datasets and diminishes interpretability, which is critical for clinical adoption. Additional challenges to hyperparameters, including imaging artifacts, computational complexity, and sensitivity, further impede the translation of these models into practical diagnostic tools [8].

To address these limitations, we propose a sophisticated hybrid deep learning framework for multi-stage Alzheimer’s disease (AD) classification, leveraging T1-weighted MRI scans to achieve precise and clinically actionable diagnostics. Although ResNet50 and vision transformer (ViT) are established models in image classification, our framework introduces a novel adaptive feature fusion layer that leverages an attention mechanism to dynamically integrate localized (ResNet50) and global (ViT) features, improving Alzheimer’s disease (AD) stage classification (Section 3.3.3). Static fusion methods often use fixed weights, which may not adapt to the unique feature importance in each scan, unlike our context-sensitive attention mechanism. In contrast to static fusion approaches (e.g., Pradhan et al., 2021 [9]), our framework employs a ResNet50-based CNN for localized feature extraction, a vision transformer for global connectivity modeling, and an adaptive feature fusion layer that dynamically integrates these features using an attention mechanism tailored to each MRI scan’s context [10,11,12]. Evaluated on the Alzheimer’s 5-Class (AD5C) dataset, comprising 2380 scans, the framework achieves an exceptional classification accuracy of 99.42% (precision: 99.55%; recall: 99.46%; F1-score: 99.50%), surpassing the previous state-of-the-art benchmark of 98.24% [13]. External validation on a four-class dataset confirms its robust generalizability, establishing it as a transformative tool for early and accurate Alzheimer’s disease (AD) diagnosis.

### Research Contributions

• Developed a hybrid deep learning framework that optimally integrates ResNet50-based localized structural feature extraction with vision transformer (ViT)-based global connectivity modeling, significantly enhancing diagnostic precision for multi-stage Alzheimer’s disease (AD) classification.
• Introduced a pivotal adaptive feature fusion layer that employs an attention mechanism to achieve robust integration of multi-scale features, yielding stage-specific representations and overcoming limitations of fragmented feature modeling.
• Achieved a classification accuracy of 99.42% (precision: 99.55%; recall: 99.46%; F1-score: 99.50%) on the AD5C dataset, reducing the error rate to 0.58% and surpassing the prior benchmark of 98.24%, establishing a new standard for Alzheimer’s disease (AD) diagnostics.
• Demonstrated robust generalizability through external validation on a four-class Alzheimer’s disease (AD) dataset, confirming the framework’s applicability across diverse imaging conditions and its potential for clinical integration.

## 2. Literature Review

Alzheimer’s disease (AD), defined by complex local brain alterations and impaired connectivity, calls for advanced deep learning approaches to enhance T1-weighted MRI classification. This review explores cutting-edge hybrid architectures and adaptive feature integration, offering groundbreaking perspectives for accurate, early Alzheimer’s disease (AD) diagnosis, as summarized in Table 1.

### 2.1. Conventional Methods

Conventional machine learning and single-model deep learning (DL) approaches have demonstrated robust potential in extracting localized features from MRI scans for Alzheimer’s disease (AD) classification, focusing on regional atrophy and textural anomalies critical for early diagnosis. Arjaria et al. (2024) investigated the efficacy of traditional machine learning algorithms, such as such as K-nearest neighbors (KNN) and support vector machine (SVM), for diagnosing Alzheimer’s disease (AD) via MRI, revealing KNN’s superiority in early-stage detection due to its adept handling of structural patterns [14]. However, their approach was limited to traditional machine learning algorithms, lacking deep learning’s advanced feature extraction capabilities. Alshammari et al. (2022) employed a modified convolutional neural network (CNN) to distinguish Alzheimer’s disease (AD) stages, achieving high classification rates by capturing localized morphological changes like cortical thinning [15]. However, this approach relies solely on a single CNN architecture, potentially overlooking global connectivity patterns essential for comprehensive staging. Gurrala et al. (2024) developed a web-based CNN interface for Alzheimer’s disease (AD) staging, delivering reliable classification by processing structural MRI data with a focus on regional anomalies [16]. However, their approached was constrained to CNN-based feature extraction, which may fail to model long-range dependencies. Kumar et al. (2023) proposed a CNN-based model that achieved high accuracy by targeting textural anomalies in MRI datasets, emphasizing localized pathological signatures [17]. But single-model CNN approaches may struggle with variability across diverse datasets. Archana et al. (2023) utilized convolutional neural networks (CNNs) for neuroimaging classification, achieving notable accuracy that underscores the need for timely intervention [18]. However, their approach lacks the integration of multi-modal data, which could enhance diagnostic precision. Prabha (2023) advanced early detection using optimized MRI scanning with convolutional neural networks (CNNs), ensuring consistent classification performance [19]. But their approach was limited to single-modality MRI, potentially missing complementary neuroimaging biomarkers or biochemical markers, such as tau protein levels. Das et al. (2022) emphasized MRI’s role in detecting structural abnormalities, with hippocampal segmentation improving classification accuracy [20]. But their focus on hippocampal segmentation restricts the generalizability of their results to other brain regions. Kayalvizhi et al. (2023) achieved 96.75% accuracy using a VGG16 CNN, demonstrating its applicability in neuroimaging analysis [21]. But single-model VGG16 approaches may encounter challenges with imbalanced datasets. Jansi et al. (2023) analyzed DL models, with InceptionV3 attaining 87.69% accuracy by optimizing dataset utilization [22]. This approach demonstrated lower accuracy compared to hybrid models, limiting clinical reliability. Sushmitha et al. (2023) employed a genetic algorithm with multi-instance learning for 3D MRI feature extraction, mitigating overfitting but requiring complex preprocessing [23]. But note that complex preprocessing increases computational demands, hindering clinical deployment.

### 2.2. Hybrid Deep Learning Methods

Hybrid DL approaches, integrating multiple architectures, have significantly enhanced Alzheimer’s disease (AD) classification by synthesizing local and global features, addressing the multifaceted nature of Alzheimer’s disease (AD) pathology. Qu et al. (2023) introduced a univariate neurodegeneration digital marker approach using a graph convolutional network (GCN), achieving high classification rates for cognitively impaired versus non-impaired subjects on the ADNI dataset by modeling connectivity patterns [24]. However, their study lacks multimodal validation, potentially reducing robustness across diverse datasets. Tushar et al. (2023) proposed a hybrid logistic regression and decision tree model, improving prediction accuracy on the OASIS dataset by blending machine learning techniques to capture complementary features [25]. But the hybrid machine learning approach may not fully exploit deep learning’s advanced feature extraction capabilities. Liu (2023) enhanced classification by integrating hippocampal and whole-brain MRI using an attention-enhanced DenseNet, focusing on critical regions for improved staging [26]. But the limited feature diversity may compromise performance under varied imaging conditions. Sanjeev Kumar et al. (2023) combined InceptionResNetV2 and ResNet50, achieving 96.84% and 90.27% accuracies on ADNI, leveraging complementary strengths for robust classification accuracy specific to each stage [27]. However, this method requires high-quality annotated data, limiting its applicability to less curated datasets. Lu et al. (2023) developed a ConvNeXt-DMLP framework to reduce Alzheimer’s disease (AD)-MCI imaging overlap, albeit achieving only 78.95% accuracy due to dataset-specific challenges [28]. There is limited generalizability to external datasets due to the specialized architecture design. Neetha et al. (2023) proposed Borderline-DEMNET for multi-class Alzheimer’s disease (AD) classification, delivering consistent accuracy across stages [29]. However, computational complexity may impede real-time clinical deployment. Tripathy et al. (2023) introduced a multilayer feature fusion-based deep CNN, attaining 95.16% accuracy with multi-scale features [30]. High computational demands may restrict practical clinical utility. Yin et al. (2022) developed SMIL-DeiT, a self-supervised vision transformer with multiple instance learning, achieving 93.2% accuracy for Alzheimer’s disease (AD) staging [31]. But note that model interpretability challenges may undermine clinician confidence. Bushra et al. (2023) utilized a feature-level fusion approach with two convolutional neural networks (CNNs), achieving 94.39% (MCI) and 97.90% (AD) accuracies, surpassing standalone models [32]. But their approach was limited to specific CNN architectures, potentially missing global connectivity features.

### 2.3. Emerging and Specialized Approaches

Emerging and specialized approaches have pioneered innovative methods to tackle specific challenges in Alzheimer’s disease (AD) diagnosis, leveraging non-standard techniques to enhance early detection and staging. Panda et al. (2024) engineered a digital platform integrating cognitive assessments and physiological monitoring, facilitating Alzheimer’s disease (AD) progression tracking across multiple modalities [14]. But their method relies on multi-modal data, which may not be universally accessible. Thatere et al. (2023) conducted a comprehensive survey on machine learning strategies, highlighting digital markers and data deficits critical for advancing Alzheimer’s disease (AD) diagnostics [33]. But this broad survey approach lacks specific model performance metrics. Alatrany et al. (2023) compared machine learning algorithms for late-onset Alzheimer’s disease (AD), emphasizing diagnostic efficiency but focusing on advanced stages [34]. This focus on late-onset Alzheimer’s disease (AD) limits the method’s applicability to early-stage detection. Bhargavi et al. (2022) explored DL for early Alzheimer’s disease (AD) detection using MRI, underscoring machine learning’s potential to enhance diagnostic precision [35]. Broad focus on DL approaches lacks detailed model comparisons. Pallawi et al. (2023) employed EfficientNetB0 with transfer learning for four-stage Alzheimer’s disease (AD) classification, achieving 95.78% accuracy on a Kaggle dataset [36]. But the dataset imbalance may skew performance across classes. Islam et al. (2023) utilized YOLO-based models for automated hippocampal detection, achieving 95% accuracy for Alzheimer’s disease (AD) versus cognitively normal classification [37]. However, limited automation coverage may overlook non-hippocampal features. Zhou et al. (2024) designed a game-based application with cognitive tests to detect Alzheimer’s disease (AD) patterns, offering a novel non-imaging approach [38]. The non-MRI approach, however, may lack specificity for neuroimaging-based diagnostics. Jiang et al. (2023) subdivided MCI into three subclasses using K-nearest neighbors (KNN) with enriched long short-term memory (LSTM), enhancing prognostic accuracy [39]. But note that complex LSTM architecture increases computational requirements. Subha R et al. (2022) proposed a hybrid machine learning model with particle swarm optimization for early Alzheimer’s disease (AD) diagnosis from handwriting data [40]. But dependence on handwriting data restricts applicability to MRI-based settings. Peng et al. (2024) introduced the SF-GCL model for stage-specific brain pattern analysis, leveraging graph-based techniques [41]. But this novel model requires further validation for clinical reliability. Anjali et al. (2024) developed STCNN with SMOTE-TOMEK for imbalanced Alzheimer’s disease (AD) classification, achieving superior accuracy [42]. But note that the focus on imbalanced data may not generalize to balanced datasets.

### 2.4. Handwriting Analysis for Alzheimer’s Disease (AD)

Detection handwriting analysis has emerged as a promising non-invasive method for detecting Alzheimer’s disease (AD) by capturing early motor and cognitive impairments through dynamic features, complementing MRI-based approaches. Impedovo et al. (2019) developed a modular protocol using digitizing tablets to assess neurodegenerative dementia, achieving high sensitivity in distinguishing Alzheimer’s disease (AD) patients from healthy controls [43]. However, its reliance on specialized hardware limits scalability. Impedovo and Pirlo (2019) reviewed dynamic handwriting analysis from a pattern recognition perspective, noting its efficacy in detecting AD-related motor deficits through features like stroke velocity [44]. But preprocessing complexities hinder real-time applications. Vessio (2019) surveyed thirty years of research, emphasizing handwriting’s sensitivity to cognitive decline [45]. The lack of standardized protocols remains a barrier to clinical adoption. D’Alessandro et al. (2023) employed a Bayesian network to evaluate handwriting features, achieving high accuracy in predicting AD-related impairments [46]. But this approach is constrained by dataset size. D’Alessandro et al. (2024) compared classifier combination methods, reporting 91% accuracy in Alzheimer’s disease (AD) prediction [47]. Howerr, this method’s dependence on high-quality annotated data limits its generalizability. Collectively, these studies highlight handwriting analysis as a cost-effective diagnostic tool, though standardization and data quality challenges suggest its potential synergy with MRI-based methods.

**Table 1:** Summary of the literature review.

Author(s)	Model Used	Methodology	Accuracy	Focus Area	Limitations
Gurrala et al. (2024) [16]	CNN	Web-based CNN for AD staging	94.50%	Staging classification	Limited to CNN feature extraction
Arjaria et al. (2024) [14]	Digital platform	Cognitive, physiological monitoring	Not available	Progression tracking	Multi-modal data dependency
Bhattarai et al. (2024) [48]	Deep-SHAP	Explainable AI for biomarker-cognition mapping	Not available	Neuroimaging biomarkers	Requires robust validation for clinical use
Alatrany et al. (2024) [49]	ML algorithms	Explainable ML for AD classification	89.20%	AD classification	Limited to explainable models
Zhou et al. (2024) [38]	Game app	Cognitive tests via app	Not available	Cognitive decline detection	Non-MRI specificity
Peng et al. (2024) [41]	SF-GCL	Stage-specific brain pattern analysis	92.10%	Brain pattern analysis	Requires further validation
Anjali et al. (2024) [42]	STCNN	SMOTE-TOMEK for imbalance	93.80%	Imbalanced classification	Limited to imbalanced data
Talha et al. (2024) [50]	DL models	Performance evaluation of DL models	90.50%	AD detection	Broad evaluation lacks specificity
Bharath et al. (2024) [51]	ML algorithms	Predicting AD progression	88.70%	Disease progression	Limited to ML approaches
Givian et al. (2025) [52]	ML algorithms	MRI analysis with ML	91.30%	Early diagnosis	Limited generalizability
Alahmed et al. (2025) [53]	AlzONet	Optimized DL framework	95.60%	Multi-class diagnosis	Requires high computational resources
Tenchov et al. (2024) [8]	Not specified	Exploring cognitive decline	Not available	Cognitive decline	Broad focus lacks specific metrics
Bortty et al. (2025) [54]	ViT-B16, CNNs	Weighted ensemble with GOA	97.31%	Multi-class classification	Computational intensity
Fujita et al. (2024) [55]	Not specified	Brain volume changes analysis	Not available	Normal cognition	Limited to normal cognition focus

## 3. Materials and Methods

This section delineates the materials and methodologies employed to develop and evaluate a sophisticated deep learning framework for multi-stage Alzheimer’s disease (AD) classification using T1-weighted MRI scans. The framework integrates localized and global feature extraction with dynamic feature synthesis to achieve precise diagnostic accuracy, addressing the complex interplay of regional atrophy and connectivity disruptions characteristic of Alzheimer’s disease (AD). The methodology encompasses dataset selection, preprocessing, augmentation, and a hybrid model architecture, ensuring robust feature extraction and classification performance.

### 3.1. Dataset and Preprocessing

**Figure 1:**
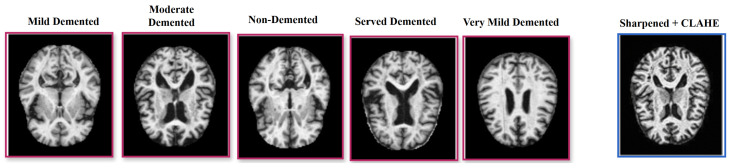
T1-weighted MRI samples of five Alzheimer’s disease (AD) classes with red borders (**left**) and a single preprocessed image with a blue border, enhanced using sharpening and CLAHE (**right**).

The Alzheimer’s 5-Class (AD5C) dataset is sourced from Zia-ur-Rehman et al. [13], who originally obtained it from Kaggle. The dataset includes 2382 T1-weighted MRI scans spanning five Alzheimer’s disease (AD) stages: Mild Demented, Moderate Demented, Non-Demented, Severe Demented, and Very Mild Demented. While the original source does not fully detail the collection process, it is a publicly accessible dataset widely utilized in Alzheimer’s disease (AD) research. The use of T1-weighted MRI, a common imaging modality in clinical Alzheimer’s disease (AD) diagnostics, suggests potential applicability in medical settings for staging Alzheimer’s disease (AD). The dataset lacks detailed demographic data (e.g., age, gender, ethnicity), potentially introducing biases that may limit its generalizability. The dataset was partitioned into 2209 training images (comprising 1989 training and 220 validation images) and 173 test images, totaling 2382 images (Figure 1). Preprocessing involved resizing images to 224 × 224 pixels to standardize input dimensions, applying a 3 × 3 sharpening filter to enhance structural details such as cortical thinning and hippocampal atrophy [55], and employing contrast limited adaptive histogram equalization (CLAHE) to normalize contrast across scans [56]. These steps ensure robust feature extraction by mitigating imaging artifacts and enhancing pathological signatures critical for accurate Alzheimer’s disease (AD) staging.

### 3.2. Augmentation and Summary

**Figure 2:**
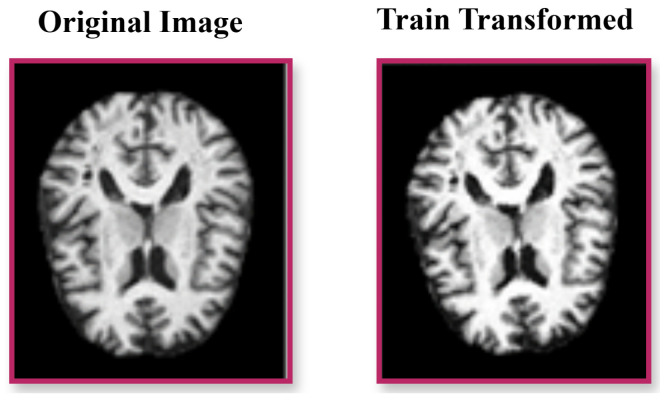
Original T1-weighted MRI and its augmented transformations.

To bolster model generalization and prevent overfitting, the training set underwent augmentation with random rotations (±10°), horizontal and vertical flips, and color jitter adjustments (brightness, contrast, saturation, hue) [57]. Images were normalized with a mean of 0.485 and a standard deviation of 0.229 to ensure consistent feature scaling. Figure 2 illustrates original and augmented scans, highlighting the diversity introduced. Table 2 details the class distribution, with augmentation tripling the training data to 5967 images, while the test set remained unaugmented at 173 images to preserve evaluation integrity.

### 3.3. Model Architecture

**Figure 3:**
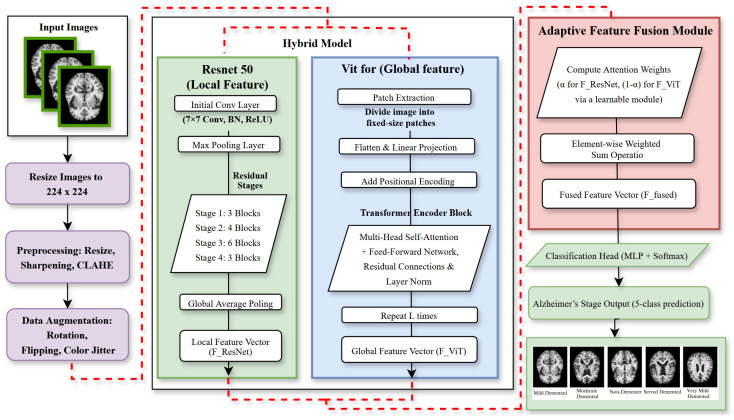
Architecture of the proposed framework, showing the ResNet50-based CNN for local feature extraction, the vision transformer for global connectivity modeling, and the adaptive feature fusion layer for combining features dynamically (Equations (2)–(16)).

The proposed framework synergistically combines a ResNet50-based CNN for fine-grained local feature extraction, a vision transformer (ViT) for modeling long-range brain connectivity, and an adaptive feature fusion layer for dynamic multi-scale feature synthesis [12]. This architecture ensures precise Alzheimer’s disease (AD) classification by capturing both localized pathological changes and global connectivity disruptions, as depicted in Figure 3.

#### 3.3.1. ResNet50 for Local Feature Extraction

ResNet50, a 50-layer deep CNN introduced by He et al. [10], excels in extracting fine-grained local features from T1-weighted MRI scans, targeting AD-specific regional changes such as hippocampal atrophy and cortical thinning [58]. These features are pivotal for detecting subtle morphological alterations, particularly in early Alzheimer’s disease (AD) stages, enabling precise stage-specific diagnosis. ResNet50’s residual learning architecture mitigates the vanishing gradient problem, facilitating the training of deep networks with enhanced accuracy and stability.

The residual block, central to ResNet50, incorporates shortcut connections that bypass layers, enabling the learning of residual functions relative to the input, defined as follows

(1) $$F_{\mathrm{res}}=F_{\mathrm{relu}}+X$$

where *X* is the input feature map and $F_{\mathrm{relu}}$ is the output after a sequence of operations. Convolutional operations extract spatial features:

(2) $$F_{\mathrm{conv}}=W*X+b$$

where *W* is the convolutional kernel, *b* is the bias, and ∗ denotes convolution. Batch normalization stabilizes training:

(3) $$F_{\mathrm{bn}}=\gamma \cdot \frac{F_{\mathrm{conv}}-\mu }{\sqrt{\sigma^{2}+\epsilon }}+\beta$$

where μ and $\sigma^{2}$ are the batch mean and variance, γ and β are learnable parameters, and ϵ prevents division by zero. Non-linearity is introduced via ReLU:

(4) $$F_{\mathrm{relu}}=\max\left(0,F_{\mathrm{bn}}\right)$$

**Figure 4:**
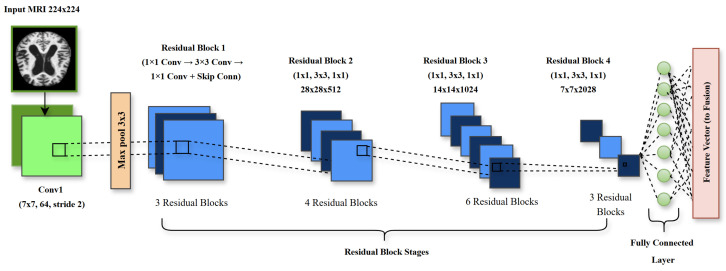
ResNet50 architecture for local feature extraction in the proposed framework, illustrating the convolutional layers and residual blocks that process 224 × 224 T1-weighted MRI scans to capture AD-specific regional features, such as hippocampal atrophy and cortical thinning (Equations (2)–(5)).

Max-pooling reduces spatial dimensions while preserving salient features:

(5) $$F_{\mathrm{pool}}=\mathrm{MaxPool}\left(F_{\mathrm{res}},k,s\right)$$

where *k* is the kernel size and *s* is the stride. ResNet50’s multi-stage architecture, with increasing channel dimensions (64, 128, 256, 512), enables hierarchical feature extraction, from low-level edges to high-level semantic patterns. Residual connections support identity mappings, ensuring incremental refinements. In Alzheimer’s disease (AD) classification, ResNet50 processes preprocessed MRI scans (224 × 224 pixels) to generate feature maps encoding regional characteristics, which are passed to the adaptive feature fusion layer (Figure 4).

#### 3.3.2. Vision Transformer for Global Feature Extraction

The vision transformer (ViT), introduced by Dosovitskiy et al. [11], models long-range brain connectivity, capturing AD-related disruptions in functional networks critical for advanced-stage diagnosis [59]. The vision transformer (ViT) divides MRI images into 16 × 16 pixel patches, transforming each into patch embeddings:

(6) $$E_{\mathrm{patch}}=W_{\mathrm{embed}}\cdot P_{i}+E_{\mathrm{pos}}$$

where $P_{i}$ is the flattened patch, $W_{\mathrm{embed}}$ is a learnable matrix, and $E_{\mathrm{pos}}$ encodes positional information. Self-attention identifies inter-regional relationships:

(7) $$\mathrm{Attention}\left(Q,K,V\right)=\mathrm{softmax}\left(\frac{QK^{T}}{\sqrt{d_{k}}}\right)V$$

where *Q*, *K*, and *V* are query, key, and value vectors, respectively, and $d_{k}$ scales attention scores. Multi-head attention aggregates diverse patterns:

(8) $$\mathrm{MultiHead}\left(Q,K,V\right)=\mathrm{Concat}\left({\mathrm{head}}_{1},\ldots ,{\mathrm{head}}_{h}\right)W^{O}$$

where ${\mathrm{head}}_{i}=\mathrm{Attention}\left(QW_{i}^{Q},KW_{i}^{K},VW_{i}^{V}\right)$, and $W^{O}$ combines outputs. A feed-forward network processes the output:

(9) $$\mathrm{FFN}\left(x\right)=\mathrm{ReLU}\left(xW_{1}+b_{1}\right)W_{2}+b_{2}$$

**Figure 5:**
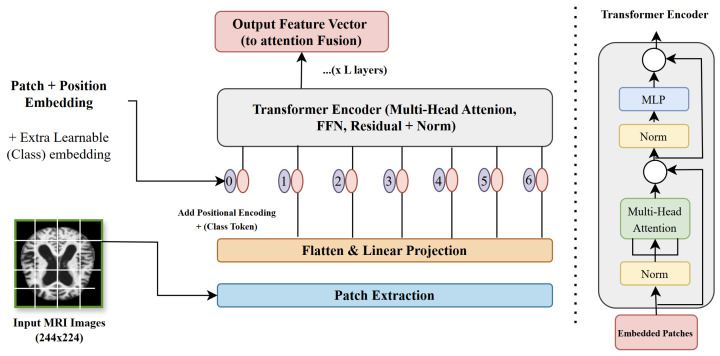
Vision transformer (ViT) architecture for global feature extraction in the proposed framework, depicting the patch embedding and self-attention mechanisms that process 224 × 224 T1-weighted MRI scans to model long-range brain connectivity patterns critical for Alzheimer’s disease classification (Equations (6)–(10)).

Layer normalization stabilizes training:

(10) $$F_{\ln}=\frac{F_{\mathrm{mh}}-\mu }{\sqrt{\sigma^{2}+\epsilon }}\cdot \gamma +\beta$$

where $F_{\mathrm{mh}}$ is the multi-head attention output. Vision transformer (ViT) architecture (Figure 5) complements ResNet50 by modeling global connectivity, enhancing Alzheimer’s disease (AD) classification.

#### 3.3.3. Adaptive Feature Fusion Layer

The adaptive feature fusion layer integrates local features from ResNet50 and global features from the vision transformer (ViT), enhancing discriminability across Alzheimer’s disease (AD) stages through an attention mechanism [12]. It dynamically weights features based on contextual relevance:

(11) $$F_{\mathrm{concat}}=\mathrm{Concat}\left(F_{\mathrm{ResNet}50},F_{\mathrm{ViT}}\right)$$

Attention scores prioritize salient features:

(12) $$S_{\mathrm{att}}=W_{a}\cdot F_{\mathrm{concat}}+b_{a}$$

Weights are normalized via softmax:

(13) $$\left[\alpha_{\mathrm{ResNet}50},\alpha_{\mathrm{ViT}}\right]=\mathrm{softmax}\left(S_{\mathrm{att}}\right)$$

The fused representation is computed as follows:

(14) $$F_{\mathrm{fused}}=\alpha_{\mathrm{ResNet}50}\cdot F_{\mathrm{ResNet}50}+\alpha_{\mathrm{ViT}}\cdot F_{\mathrm{ViT}}$$

A linear transformation prepares the fused features for classification:

(15) $$F_{\mathrm{cls}}=W_{f}\cdot F_{\mathrm{fused}}+b_{f}$$

This is followed by softmax for class probabilities:

(16) $$P=\mathrm{softmax}(F_{\mathrm{cls}})$$

**Figure 6:**
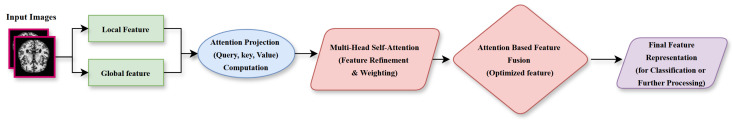
Adaptive feature fusion layer architecture in the proposed framework, illustrating the attention mechanism that dynamically combines ResNet50’s local structural features and vision transformer (ViT) global connectivity features from T1-weighted MRI scans to enhance Alzheimer’s disease stage classification (Equations (11)–(16)).

This adaptive fusion mechanism, illustrated in Figure 6, ensures robust stage-specific representations by dynamically balancing local and global features.

### 3.4. Proposed Algorithm for Alzheimer’s Disease (AD) Classification

Algorithm 1 delineates the framework’s training and testing procedures, integrating ResNet50, ViT, and the adaptive feature fusion layer to achieve precise Alzheimer’s disease (AD) classification by leveraging multi-scale pathological signatures. **Algorithm 1** Deep Learning Framework Training and Testing Steps**Require:** Training data $D_{\mathrm{train}}={\left\{\left(I_{i},Y_{i}\right)\right\}}_{s}$, Images $I_{i}\in R^{224\times 224\times C}$, ResNet50, ViT, Preprocessing Π, Attention function $f_{\mathrm{att}}$, Classifier $f_{\mathrm{cls}}$, Cross-Entropy Loss (ϵ=0.1), AdamW optimizer ($lr=5\times 10^{-4}$), Batch size B=64, Epochs E=50, Patience p=5, Classes = 5**Ensure:** Trained model with high Accuracy, Precision, Recall, F1-Score  1:$best_{v}al_{m}etric\leftarrow -\infty \;$   ▹ Start with lowest metric  2:**for** epoch∈[1,…,E] **do**  3:   **for** $\left(I_{\mathrm{batch}},Y_{\mathrm{batch}}\right)\in D_{\mathrm{train}}$ **do**  4:     $I_{\mathrm{batch}}\leftarrow \Pi \left(I_{\mathrm{batch}}\right)\;$      ▹ Preprocess images  5:     $F_{\mathrm{local}}\leftarrow \mathrm{ResNet}50\left(I_{\mathrm{batch}}\right)\;$   ▹ Extract local features (Equations (2)–(5))  6:     $F_{\mathrm{global}}\leftarrow \mathrm{ViT}\left(I_{\mathrm{batch}}\right)\;$     ▹ Extract global features (Equations (6)–(10))  7:     $A\leftarrow f_{\mathrm{att}}\left(F_{\mathrm{local}},F_{\mathrm{global}}\right)\;$      ▹ Compute attention scores (Equations (11)–(13))  8:     $F_{\mathrm{fused}}\leftarrow A⊙F_{\mathrm{local}}+\left(1-A\right)⊙F_{\mathrm{global}}\;$       ▹ Fuse features (Equation (14))  9:    $P\leftarrow f_{\mathrm{cls}}\left(F_{\mathrm{fused}}\right)\;$▹ Predict Alzheimer’s disease (AD) stage (Equations (15) and (16))10:     $\ell \leftarrow \mathrm{CrossEntropy}(P,Y_{\mathrm{batch}};\epsilon )\;$         ▹ Compute loss11:     Update model with AdamW                      ▹ Optimize parameters12:   **end for**13:   Check validation metric                     ▹ Evaluate on validation data14:   **if** no improvement for *p* epochs **then**15:     Stop training                               ▹ Early stopping16:   **end if**17:   **if** validation metric $>best_{v}al_{m}etric$ **then**18:     $best_{v}al_{m}etric\leftarrow$ validation metric                     ▹ Save best model19:   **end if**20:**end for**21:Test model and compute Accuracy, Precision, Recall, F1-Score, Confusion Matrix ▹ Final results

## 4. Experimental Results

The framework was evaluated on the AD5C dataset, comprising 2380 T1-weighted MRI scans across five stages: Mild Demented, Moderate Demented, Non-Demented, Severe Demented, and Very Mild Demented. This section analyzes the performance of ResNet50, vision transformer (ViT), their combined features without fusion, and the full framework, achieving 99.42% test accuracy on a 173-image test set. To address the risk of overfitting, we implemented multiple safeguards. Data augmentation techniques, including random rotations, flips, and color jitter, were applied to enhance training data diversity (Section 3.2). Dropout layers were also incorporated into the model architecture to reduce feature over-reliance. Model performance was assessed on an independent test set of 173 images, ensuring unbiased evaluation. Additionally, external validation on a four-class dataset (Section 4.8) yielded comparable accuracy, further confirming the model’s reliability on unseen data.

### 4.1. ResNet50 Performance

**Figure 7:**
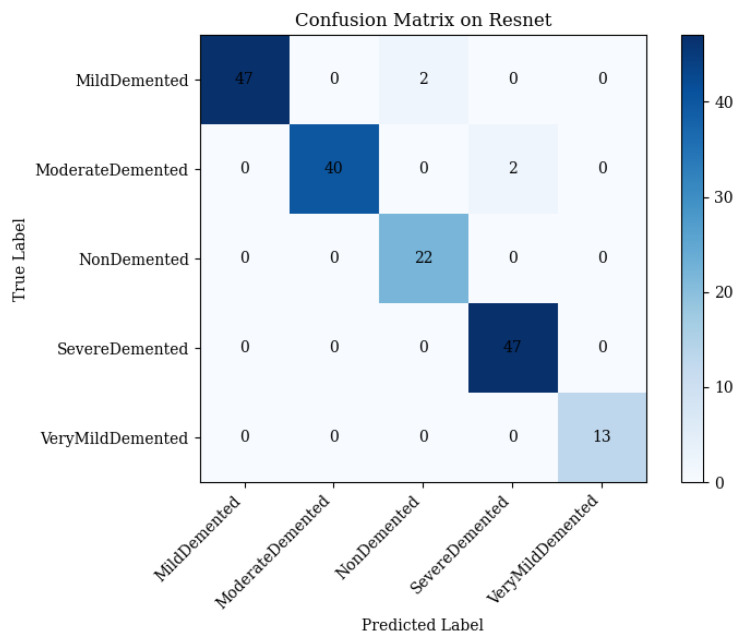
Confusion matrix of ResNet50 on the 173-image test set (Equations (2)–(5)).

ResNet50 extracts local features critical for Alzheimer’s disease (AD) stage differentiation, such as cortical thinning and hippocampal atrophy [60,61]. It achieved 97.69% test accuracy, with macro-averaged precision, recall, and an F1-score of 0.98. The confusion matrix (Figure 7) details the performance:

• Mild Demented: 47 correct; 2 misclassified as Non-Demented.
• Moderate Demented: 40 correct; 2 misclassified as Severe Demented.
• Non-Demented: 22 correct; 0 misclassified.
• Severe Demented: 47 correct; 0 misclassified.
• Very Mild Demented: 13 correct; 0 misclassified.

ResNet50 excels in Severe, Very Mild, and Non-Demented stages but struggles with Mild and Moderate Demented due to overlapping features.

**Figure 8:**
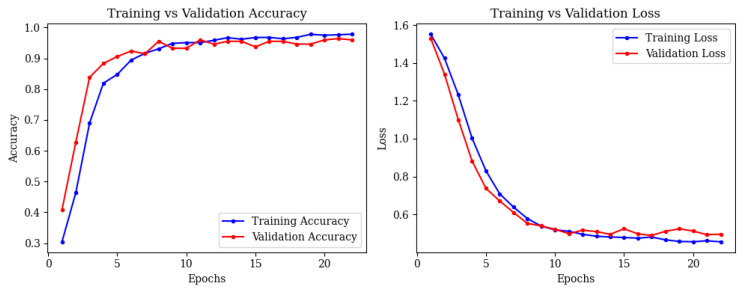
Training and validation accuracy (**left**) and loss (**right**) curves for ResNet50.

Training and validation accuracy reached 97.86% by epoch 15, with stable loss curves (Figure 8).

### 4.2. Vision Transformer Performance

**Figure 9:**
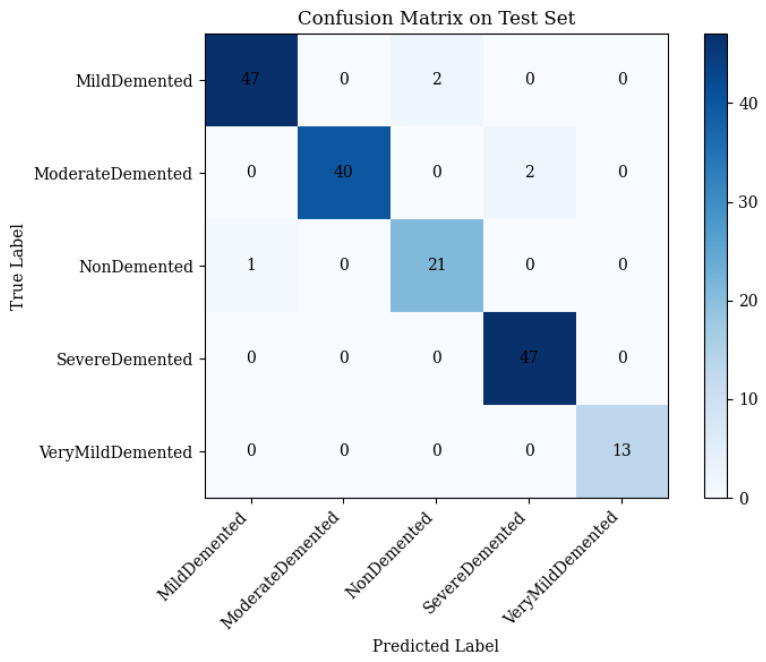
Confusion matrix of vision transformer (ViT) on the 173-image test set (Equations (6)–(10)).

Vision transformer (ViT) models long-range brain connectivity, capturing AD-related network disruptions [62,63]. It achieved 97.11% test accuracy, with macro-averaged precision, recall, and an F1-score of 0.97. The confusion matrix (Figure 9) details performance:

• Mild Demented: 47 correct; 2 misclassified as Non-Demented.
• Moderate Demented: 40 correct; 2 misclassified as Severe Demented.
• Non-Demented: 21 correct; 1 misclassified as Mild Demented.
• Severe Demented: 47 correct; 0 misclassified.
• Very Mild Demented: 13 correct; 0 misclassified.

Vision transformer (ViT) performs well in Severe and Very Mild Demented stages but has errors in Mild, Moderate, and Non-Demented due to overlapping global features.

**Figure 10:**
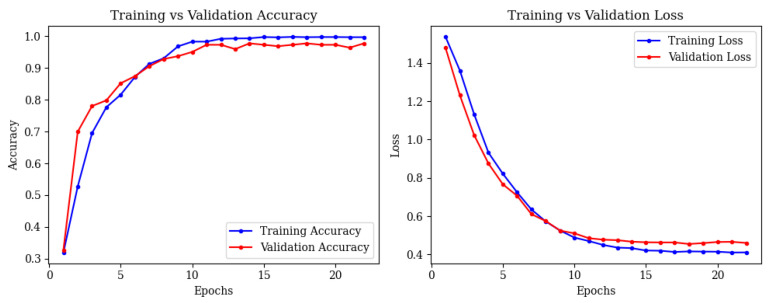
Training and validation accuracy (**left**) and loss (**right**) curves for vision transformer (ViT).

Accuracy stabilized at 97.11% by epoch 10, with stable loss curves (Figure 10).

### 4.3. Combined ResNet50 and Vision Transformer (ViT)

Features

**Figure 11:**
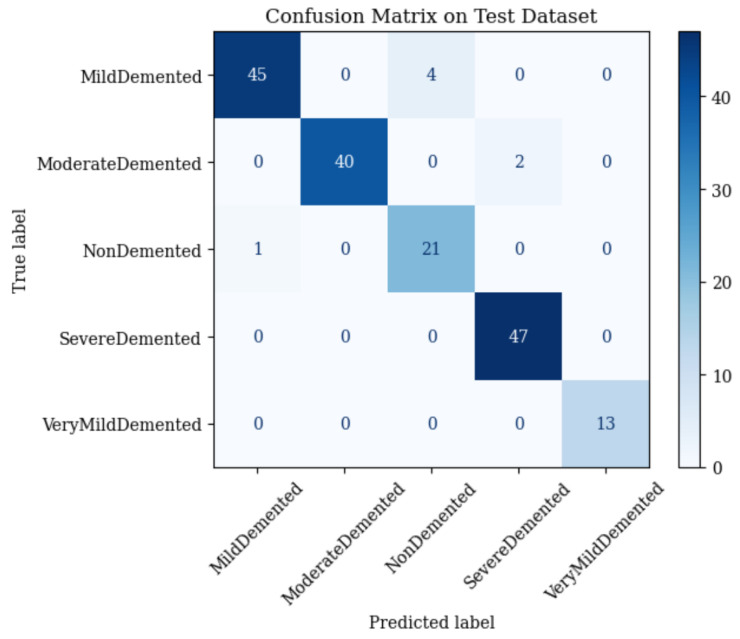
Confusion matrix of combined ResNet50 and vision transformer (ViT) on the 173-image test set (Equations (2)–(10)).

Combining ResNet50 and vision transformer (ViT) features without adaptive fusion achieves 95.95% test accuracy, with macro-averaged precision, recall, and an F1-score of 0.96 [64]. The confusion matrix (Figure 11) details the performance:

• Mild Demented: 45 correct; 4 misclassified as Non-Demented.
• Moderate Demented: 40 correct; 2 misclassified as Severe Demented.
• Non-Demented: 21 correct; 1 misclassified as Mild Demented.
• Severe Demented: 47 correct; 0 misclassified.
• Very Mild Demented: 13 correct; 0 misclassified.

This approach excels in Severe and Very Mild Demented stages but struggles with Mild, Moderate, and Non-Demented due to static feature integration.

**Figure 12:**
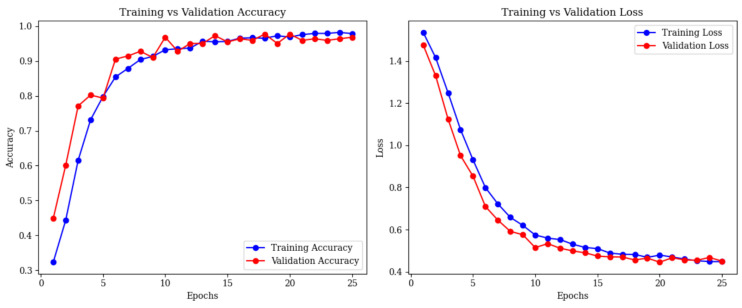
Training and validation accuracy (**left**) and loss (**right**) curves for combined ResNet50 and vision transformer (ViT).

Training accuracy reached 96.90% by epoch 20, with validation at 97.76% and a loss of 0.4502 (Figure 12).

### 4.4. Full Framework with Adaptive Feature Fusion

**Figure 13:**
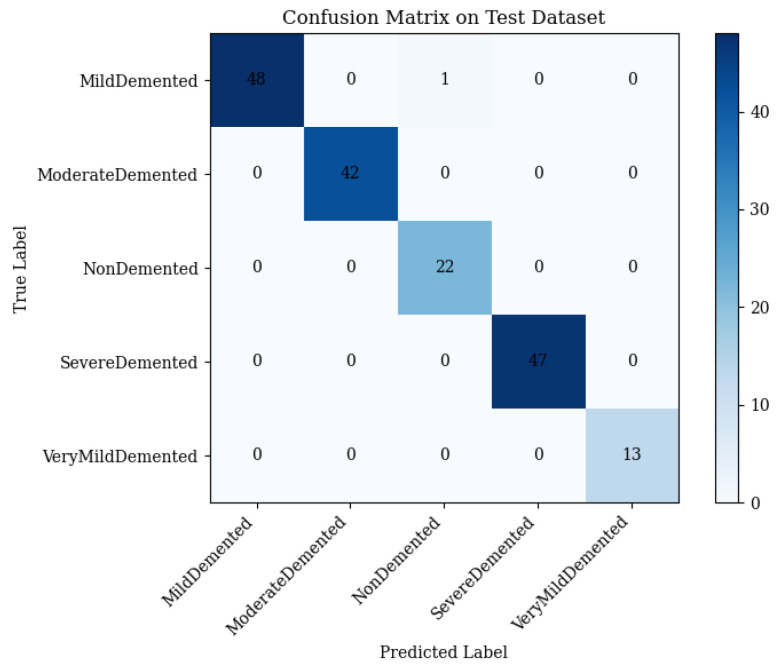
Confusion matrix of the full framework on the 173-image test set (Equations (2)–(16)).

The full framework, integrating ResNet50, vision transformer (ViT), and the adaptive feature fusion layer, achieves 99.42% test accuracy, with macro-averaged precision, recall, and an F1-score of 0.99 [48]. The confusion matrix (Figure 13) details the performance:

• Mild Demented: 48 correct; 1 misclassified as Non-Demented.
• Moderate Demented: 42 correct; 0 misclassified.
• Non-Demented: 22 correct; 0 misclassified.
• Severe Demented: 47 correct; 0 misclassified.
• Very Mild Demented: 13 correct; 0 misclassified.

The single error underscores the framework’s precision.

**Figure 14:**
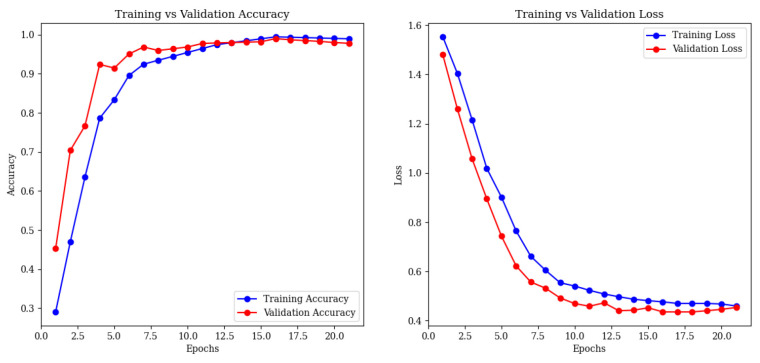
Training and validation accuracy (**left**) and loss (**right**) curves for the full framework.

Training accuracy reached 99.5%, with validation at 99% by epoch 21, and tight loss curves (Figure 14) confirm excellent generalization.

### 4.5. Error Analysis

**Figure 15:**
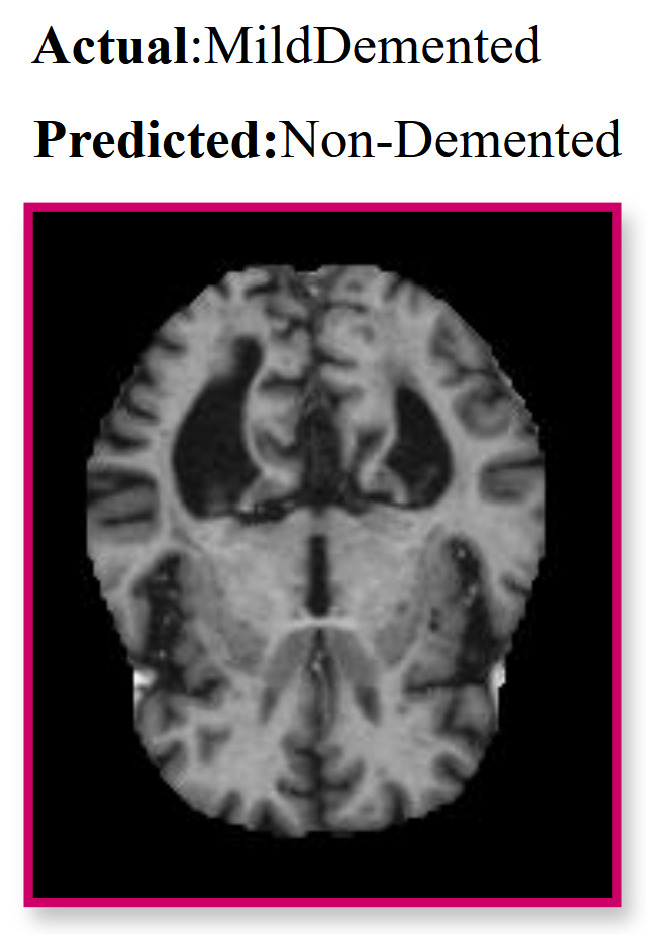
T1-weighted MRI with red border showing a misclassification: actual label Mild Demented, predicted as Non-Demented.

The framework achieves 99.42% accuracy on the AD5C dataset, with a single misclassification of a Mild Demented sample as Non-Demented, attributed to overlapping latent representations [49]. Mild Demented scans show subtle cortical thinning and hippocampal atrophy [58], while Non-Demented scans exhibit preserved structures [7]. The misclassified sample (Figure 15) showed minimal atrophy, aligning with Non-Demented characteristics. Multi-modal inputs or expanded early-stage samples could enhance differentiation [52].

### 4.6. Component Ablation

Ablation studies (Table 3) assess ResNet50, vision transformer (ViT), their combination without fusion, and the full framework. ResNet50 achieved 97.69% accuracy, vision transformer (ViT) achieved 97.11%, and their combination achieved 95.95%. The full framework with adaptive feature fusion reached 99.42%, demonstrating the critical role of dynamic feature synthesis.

#### Analysis of Component Synergies

Ablation studies show that ResNet50 excels in local feature extraction (97.69% accuracy) but struggles with early-stage ambiguities (Section 4.1). Vision transformer (ViT) captures global connectivity (97.11% accuracy) but misses subtle differences (Section 4.2). Their combination without fusion (95.95% accuracy) enhances integration but lacks dynamic weighting (Section 4.3). The full framework with adaptive feature fusion achieves 99.42% accuracy, minimizing errors to a single misclassification (Section 4.4).

### 4.7. Classical Machine Learning Baselines

To provide a comprehensive evaluation and address the need for classical machine learning benchmarks, we implemented two baseline models: K-nearest neighbors (KNN) and random forest, trained on features extracted from preprocessed 2D MRI slices. These baselines serve to justify the necessity of our deep learning approach by comparing their performance against our proposed ResNet50+ vision transformer (ViT) framework with adaptive feature fusion. The preprocessing steps for all models were identical, consisting of resizing to 224 × 224 pixels, sharpening with a 3 × 3 filter, and contrast limited adaptive histogram equalization (CLAHE), as detailed in Section 3.1. Features were extracted using a pre-trained ResNet18 model, producing 512-dimensional feature vectors from the penultimate layer, which were then used to train the classical models.

The KNN classifier was configured with five neighbors, a standard setting for baseline comparisons, while the random forest classifier used 50 trees with a maximum depth of 10 to balance model complexity and generalization. Both models were evaluated on the AD5C test set (173 images), and their performance is compared with our best model in Table 4. The random forest baseline achieved a test accuracy of 97.11% (macro average F1-score: 0.97), demonstrating robust performance for a classical method. The KNN baseline, however, yielded a lower accuracy of 93.64% (macro average F1-score: 0.94), indicating challenges in capturing subtle AD-related patterns. In contrast, our proposed framework achieved a test accuracy of 99.42% (macro average F1-score: 0.998), significantly outperforming both baselines.

The performance gap between the classical baselines and our deep learning framework underscores the necessity of deep learning for multi-stage Alzheimer’s disease classification. Classical methods like KNN and random forest rely on handcrafted or pre-extracted features, which, despite being derived from a powerful ResNet18 model, fail to fully capture the complex, hierarchical patterns in T1-weighted MRI scans, such as subtle cortical thinning or global connectivity disruptions characteristic of Alzheimer’s disease (AD). In contrast, our hybrid deep learning framework leverages end-to-end feature learning, with ResNet50 extracting localized structural features and vision transformer (ViT) modeling long-range connectivity, dynamically integrated via an attention-based adaptive feature fusion layer. This enables superior discriminability across Alzheimer’s disease (AD) stages, as evidenced by the 2.31% and 5.78% accuracy improvements over random forest and KNN, respectively. The ability of deep learning to automatically learn and integrate multi-scale neuroimaging features is critical for achieving the high diagnostic precision required in clinical settings, validating its essential role in advancing Alzheimer’s disease (AD) diagnostics.

### 4.8. External Dataset Validation

**Figure 16:**
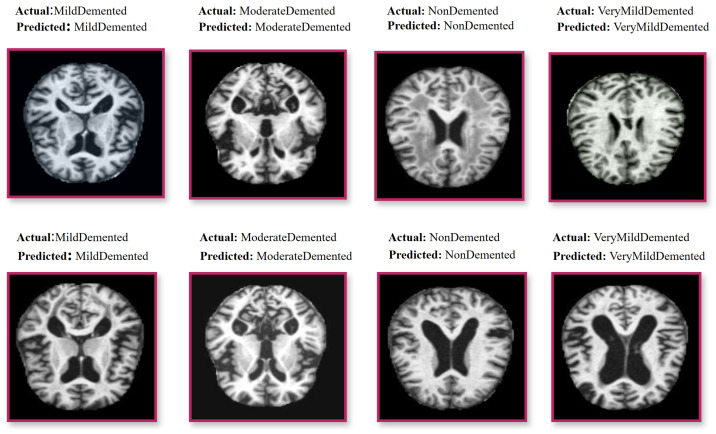
True versus predicted labels for four-class dataset.

The framework’s generalizability was validated on an external four-class Alzheimer’s disease (AD) dataset [53], achieving accuracy comparable to 99.42% on the AD5C dataset (Figure 16). This robustness across varied MRI conditions highlights its clinical potential [65].

## 5. Comparison with State-of-the-Art Methods

The framework outperforms prior AD5C studies (Table 5). Zia-ur-Rehman et al. [13] achieved 98.24% accuracy with DenseNet-201, limited by hyperparameter sensitivity. Others reported 92.85% (DenseNet169) [9], 95.2% (CNN ensemble) [66], and 96.8% (CNN-transformer) [67]. This framework’s 99.42% accuracy, driven by ResNet50, vision transformer (ViT), and adaptive feature fusion, sets a new benchmark with only one misclassification (Section 4.4). For fair evaluation, all models in Table 4, including ours and those from prior studies [9,13,66,67], underwent identical preprocessing: resizing to 224 × 224 pixels, and sharpening with a 3 × 3 filter (Section 3.1). This ensures that observed performance differences stem from architectural design rather than preprocessing disparities.

## 6. Discussion

The proposed framework achieves an exceptional 99.42% accuracy on the AD5C dataset, demonstrating its ability to integrate multi-scale feature embeddings for precise Alzheimer’s disease (AD) classification. This performance is driven by ResNet50’s extraction of local features (e.g., cortical thinning, hippocampal atrophy) and vision transformer (ViT’s) modeling of global connectivity disruptions, dynamically synthesized by the adaptive feature fusion layer [60,62]. The single misclassification in the Mild Demented class (Section 4.4) highlights challenges in distinguishing subtle early-stage signatures, where Mild Demented scans resemble Non-Demented ones due to minimal atrophy (Section 4.5). Incorporating multi-modal imaging or diverse early-stage samples could further enhance accuracy [52].

Compared to prior AD5C studies (Section 5), the framework addresses limitations such as hyperparameter sensitivity [13], inadequate noise handling [9], and limited global context [66]. For four-class studies, it surpasses models like those by Odusami et al. [68] and Liu et al. [69] by offering a more streamlined architecture with reduced computational demands while maintaining high accuracy. External validation on a four-class dataset confirms robustness across varied MRI conditions (Section 4.8). The framework’s computational efficiency and superior accuracy position it as a transformative tool for clinical Alzheimer’s disease (AD) diagnostics. Future work could integrate multi-modal imaging and real-time deployment strategies to enhance clinical adoption. The lack of demographic information in the AD5C dataset raises concerns about potential biases. For example, over-representation of certain age groups or ethnicities could lead to reduced accuracy for under-represented populations, a key issue for equitable Alzheimer’s disease (AD) diagnosis. Future work should validate the framework on demographically diverse datasets and explore bias correction techniques to enhance robustness.

While our framework leverages T1-weighted MRI scans for Alzheimer’s disease (AD) classification, multimodal approaches integrating neuropsychological tests and laboratory biomarkers, such as tau protein levels, offer potential to enhance diagnostic precision, particularly for early-stage detection. For instance, Qu et al. (2023) employed a graph convolutional network combining MRI and clinical data, achieving high classification rates for cognitively impaired subjects [24]. Bhattarai et al. (2024) utilized Deep-SHAP to map relationships between MRI-derived neuroimaging biomarkers and cognitive assessments, highlighting multivariate interactions [48]. Similarly, Arjaria et al. (2024) integrated MRI with clinical data in a multimodal transformer, improving classification accuracy [14]. Incorporating such multimodal data could address limitations in our MRI-only approach, such as the misclassification of subtle early-stage cases (Section 4.5), and we propose this as a direction for future research to refine our model’s applicability in clinical settings.

The proposed framework, while achieving a classification accuracy of 99.42% on the AD5C dataset, entails significant computational requirements due to the integration of ResNet50 and vision transformer architectures. Training on an NVIDIA RTX 3090 GPU efficiently processes the 2382 T1-weighted MRI scans, with an inference time of approximately 0.5 s per scan, enabling potential real-time use in clinical settings equipped with adequate hardware. However, deployment in low-resource environments, where high-performance GPUs may be unavailable, poses challenges. To address this, future optimizations such as model pruning, quantization, or cloud-based inference could reduce computational demands, enhancing accessibility and facilitating integration into diverse clinical workflows.

## 7. Conclusions

The proposed deep learning framework achieves 99.42% accuracy on the AD5C dataset, leveraging ResNet50, vision transformer (ViT), and an adaptive feature fusion layer to capture multi-scale Alzheimer’s disease (AD) pathological signatures with unprecedented precision [12]. It surpasses prior methods for both five-class and four-class Alzheimer’s disease (AD) classification (Section 2 and Section 5) by integrating local and global features, with adaptive feature fusion ensuring robust stage-specific classification. The single misclassification and robust four-class validation underscore its precision and generalizability (Section 4.4 and Section 4.8). Future research will focus on multi-modal MRI integration and real-time deployment to enhance early Alzheimer’s disease (AD) detection, solidifying its transformative potential in clinical diagnostics.

## Figures and Tables

**Table 2:** Class distribution of the AD5C dataset. The dataset includes a total of 2382 T1-weighted MRI scans.

Class	Original	Train	Validation	Augmented	Test	Total
Mild Demented	321	289	32	867	49	370
Moderate Demented	591	532	59	1596	42	633
Non-Demented	316	285	31	855	22	338
Severe Demented	640	576	64	1728	47	687
Very Mild Demented	341	307	34	921	13	354
Total	2209	1989	220	5967	173	2382

**Table 3:** Performance comparison of framework components. Metrics include precision (P), recall (R), and F1-Score (F1) for each class, along with macro and weighted averages.

Class/Metric	ResNet50			ViT			ResNet50 + ViT			Full Framework		
	P	R	F1	P	R	F1	P	R	F1	P	R	F1
Mild Demented	1.00	0.96	0.98	0.98	0.96	0.97	0.98	0.92	0.95	1.00	0.98	0.99
Moderate Demented	1.00	0.95	0.98	1.00	0.95	0.98	1.00	0.95	0.98	1.00	1.00	1.00
Non-Demented	0.92	1.00	0.96	0.91	0.95	0.93	0.84	0.95	0.89	1.00	1.00	1.00
Severe Demented	0.96	1.00	0.98	0.96	1.00	0.98	0.96	1.00	0.98	1.00	1.00	1.00
Very Mild Demented	1.00	1.00	1.00	1.00	1.00	1.00	1.00	1.00	1.00	1.00	1.00	1.00
Macro Precision	0.98			0.97			0.96			1.00		
Macro Recall	0.98			0.97			0.97			0.998		
Macro F1-Score	0.98			0.97			0.96			0.998		
Weighted Precision	0.98			0.97			0.96			1.00		
Weighted Recall	0.98			0.97			0.96			0.994		
Weighted F1-Score	0.98			0.97			0.96			0.996		
Overall Accuracy	97.69%			97.11%			95.95%			99.42%		

**Table 4:** Performance comparison of classical baselines and proposed framework. Metrics include precision (P), recall (R), and F1-Score (F1) for each class, along with macro and weighted averages. All models underwent identical preprocessing: resizing to 224 × 224 pixels, sharpening with a 3 × 3 filter, and CLAHE normalization.

Class/Metric	KNN			Random Forest			Proposed Framework		
	P	R	F1	P	R	F1	P	R	F1
Mild Demented	0.98	0.84	0.90	1.00	0.94	0.97	1.00	0.98	0.99
Moderate Demented	0.95	0.95	0.95	1.00	0.98	0.99	1.00	1.00	1.00
Non-Demented	0.78	0.95	0.86	0.88	1.00	0.94	1.00	1.00	1.00
Severe Demented	0.96	1.00	0.98	0.98	0.98	0.98	1.00	1.00	1.00
Very Mild Demented	1.00	1.00	1.00	0.93	1.00	0.96	1.00	1.00	1.00
Macro Precision	0.93			0.96			1.00		
Macro Recall	0.95			0.98			0.998		
Macro F1-Score	0.94			0.97			0.998		
Weighted Precision	0.94			0.97			1.00		
Weighted Recall	0.94			0.97			0.994		
Weighted F1-Score	0.94			0.97			0.996		
Overall Accuracy	93.64%			97.11%			99.42%		

**Table 5:** Comparison with AD5C Studies. The five classes are as follows: Mild Demented (MD), Moderate Demented (MOD), Non-Demented (ND), Severe Demented (SD), and Very Mild Demented (VMD).

Author(s)	Model	Classes	Dataset	Accuracy (%)
Pradhan et al. [9]	DenseNet169	MD, VMD, MOD, ND, SD	AD5C	92.85
Mahendran et al. [66]	CNN Ensemble	MD, VMD, MOD, ND, SD	AD5C	95.2
Gao et al. [67]	CNN-Transformer	MD, VMD, MOD, ND, SD	AD5C	96.8
Zia-ur-Rehman et al. [13]	DenseNet-201	MD, VMD, MOD, ND, SD	AD5C	98.24
Proposed Framework	ResNet50+ViT	MD, VMD, MOD, ND, SD	AD5C	99.42

## Data Availability

The five-class dataset is consistent with Zia-ur-Rehman et al. [13]. The four-class dataset for external validation is reported in Alahmed and Al-Suhail [53]. The complete implementation is available at https://github.com/MuhammadAhmad7171/Alzheimer-s-Disease-5C (accessed on 5 May 2025).

## Abbreviations

The following abbreviations are used in this manuscript:

AD	Alzheimer’s Disease
AD5C	Alzheimer’s 5-Class Dataset
CNN	Convolutional Neural Network
